# Investigating the Combinatory Effects of Biological Networks on Gene Co-expression

**DOI:** 10.3389/fphys.2016.00160

**Published:** 2016-05-02

**Authors:** Cheng Zhang, Sunjae Lee, Adil Mardinoglu, Qiang Hua

**Affiliations:** ^1^State Key Laboratory of Bioreactor Engineering, East China University of Science and TechnologyShanghai, China; ^2^Science for Life Laboratory, KTH-Royal Institute of TechnologyStockholm, Sweden; ^3^Department of Biology and Biological Engineering, Chalmers University of TechnologyGöteborg, Sweden; ^4^Shanghai Collaborative Innovation Center for Biomanufacturing TechnologyShanghai, China

**Keywords:** *Saccharomyces cerevisiae*, co-expression, co-regulation, transcriptional regulatory network, protein-protein interaction network

## Abstract

Co-expressed genes often share similar functions, and gene co-expression networks have been widely used in studying the functionality of gene modules. Previous analysis indicated that genes are more likely to be co-expressed if they are either regulated by the same transcription factors, forming protein complexes or sharing similar topological properties in protein-protein interaction networks. Here, we reconstructed transcriptional regulatory and protein-protein networks for *Saccharomyces cerevisiae* using well-established databases, and we evaluated their co-expression activities using publically available gene expression data. Based on our network-dependent analysis, we found that genes that were co-regulated in the transcription regulatory networks and shared similar neighbors in the protein-protein networks were more likely to be co-expressed. Moreover, their biological functions were closely related.

## Introduction

Recent advances involving high-throughput measurements have facilitated the accumulation of extensive gene expression profile sets, and the gene expression patterns evident across multiple conditions have allowed a systems-level understanding of biology. Specifically, gene co-expression has been proposed as a useful methodology for uncovering gene functions (Stuart et al., 2003), and this approach prevails in many areas of systems biology (Carter et al., 2004; Aoki et al., 2007; Miller et al., 2008, 2010; Presson et al., 2008; Xulvi-Brunet and Li, 2010; Liao et al., 2011; MacDonald et al., 2015; Peake et al., 2015).

If the mRNA expression levels of two genes follow a similar pattern through multiple gene expression measurements, they are treated as co-expressed genes. Gene co-expression indicates that the transcription rates of these genes are modulated by similar molecules, and this underlying modularity has allowed us to uncover the functions of co-expressed genes. Transcription factors (TFs) bind to the promoters of target genes and this kind of binding modulates their expression at the mRNA level. Under different physiological conditions, two or more TFs can bind to the same target gene (Balaji et al., 2006), and this phenomenon is known as combinatorial regulation. Through combinatorial binding, a small set of TFs can dynamically modulate gene expression in response to diverse physiological conditions.

In the past decade, a large amount of high-throughput gene expression data has been made publically available, and this information has facilitated the development of global co-expression analyses. Consequently, various methods and algorithms have been developed for generating gene co-expression networks (Bar-Joseph et al., 2003; Tong et al., 2004; Zhang and Horvath, 2005; Luo et al., 2007; Langfelder and Horvath, 2008; Ruan et al., 2010; Savage et al., 2010; Roy et al., 2014).

Although co-expression analysis had been widely used, knowledge of the biological factors and topological characteristics that contribute to high gene co-expression remains limited. Currently, the knowledge of regulatory networks is increasing (Teixeira et al., 2006, 2014; Monteiro et al., 2008; Abdulrehman et al., 2011; Hughes and de Boer, 2013), but the combinatorial regulation by TFs makes it difficult to understand the contributing factors in co-expression. Through hundreds of expression measurements, a genome-scale study demonstrated that genes bound by similar TFs are highly co-expressed (Allocco et al., 2004). In addition, the expression profiles of genes coding for interacting protein pairs are more highly correlated than random pairs (Ge et al., 2001), but later genome-scale studies indicated that this correlation was significantly reduced and suggested that only those ones forming the same protein complex seem to be co-expressed (Bhardwaj and Lu, 2005; Xulvi-Brunet and Li, 2010). Furthermore, the topological properties of the protein coding genes in protein-protein interaction (PPI) networks may also contribute to the co-expression of genes (Han et al., 2004).

Although efforts have been made to explore the relationship between co-expression and biological networks, to the best of our knowledge, no study had exclusively analyzed the joint effects of different biological networks [e.g., transcriptional regulatory (TR) and protein–protein interactions (PPI) networks] on gene co-expression. In this study, we reconstructed TR and PPI networks based on well-established databases for biological interactions in *Saccharomyces cerevisiae*. We investigated the relationships between gene co-expression in each network as well as in multiple biological networks. Moreover, we constructed co-regulated biological networks and comprehensively studied their effects on gene co-expression.

## Materials and methods

### Microarray data

Gene expression data for *S. cerevisiae* were retrieved from the Gene Expression Omnibus (GEO) database. All microarray data for *S. cerevisiae* using GPL2529 as platform and published before January 22th, 2014 in GEO database were selected. And to eliminate replicate size-based biases, only one replicate (replicate 1) were included in the analysis when multiple replicates are available. As a result, 1057 microarray datasets were selected. The microarray data were normalized with the Robust Multiarray Average (RMA; Bolstad et al., 2003; Irizarry et al., 2003a,b) and treated with the affy R package (Gautier et al., 2004). Open reading frame (ORF) ids were converted to gene ids. If a gene was mapped with more than one ORF, the mean value was used in our analysis. Consequently, expression profiles for 5657 genes in 1057 different experimental conditions were obtained (Table S1). Finally, the pair-wise gene co-expression data were obtained for 5657 genes by calculating the Pearson correlation coefficients using MATLAB R2015a.

### Reconstruction of regulatory networks

TR interactions for *S. cerevisiae* were retrieved from the YEASTRACT database (Teixeira et al., 2006, 2014; Monteiro et al., 2008; Abdulrehman et al., 2011). Two evidence types were presented in YEASTRACT: regulation with DNA binding evidence and regulation with expression evidence (i.e., expression evidence from TF knock-out or over-expression experiments). In this study, we treated these two regulation types independently. Consequently, we reconstructed two networks: a regulatory network with only the DNA binding evidence (regardless of the expression evidence), hereafter referred to as Bnet, and a regulatory network with only expression evidence (regardless of the binding evidence), hereafter referred to as Enet (Figure S1). Regulation data pertaining to genes that are not included in the microarray data were eliminated from the reconstructed networks in this study.

### Reconstruction of a protein-protein interaction network

BioGRID is a database that includes comprehensive information about PPIs from diverse organisms (Stark et al., 2006; Chatr-aryamontri et al., 2013, 2015). All available PPI information for *S. cerevisiae* was retrieved from BioGRID, and a PPI network was reconstructed, hereafter referred to as Pnet. Similarly, interactions with genes that are not included in the microarray data were eliminated from the reconstructed Pnet.

### Construction of “co-regulated” networks

**Figure 1:**
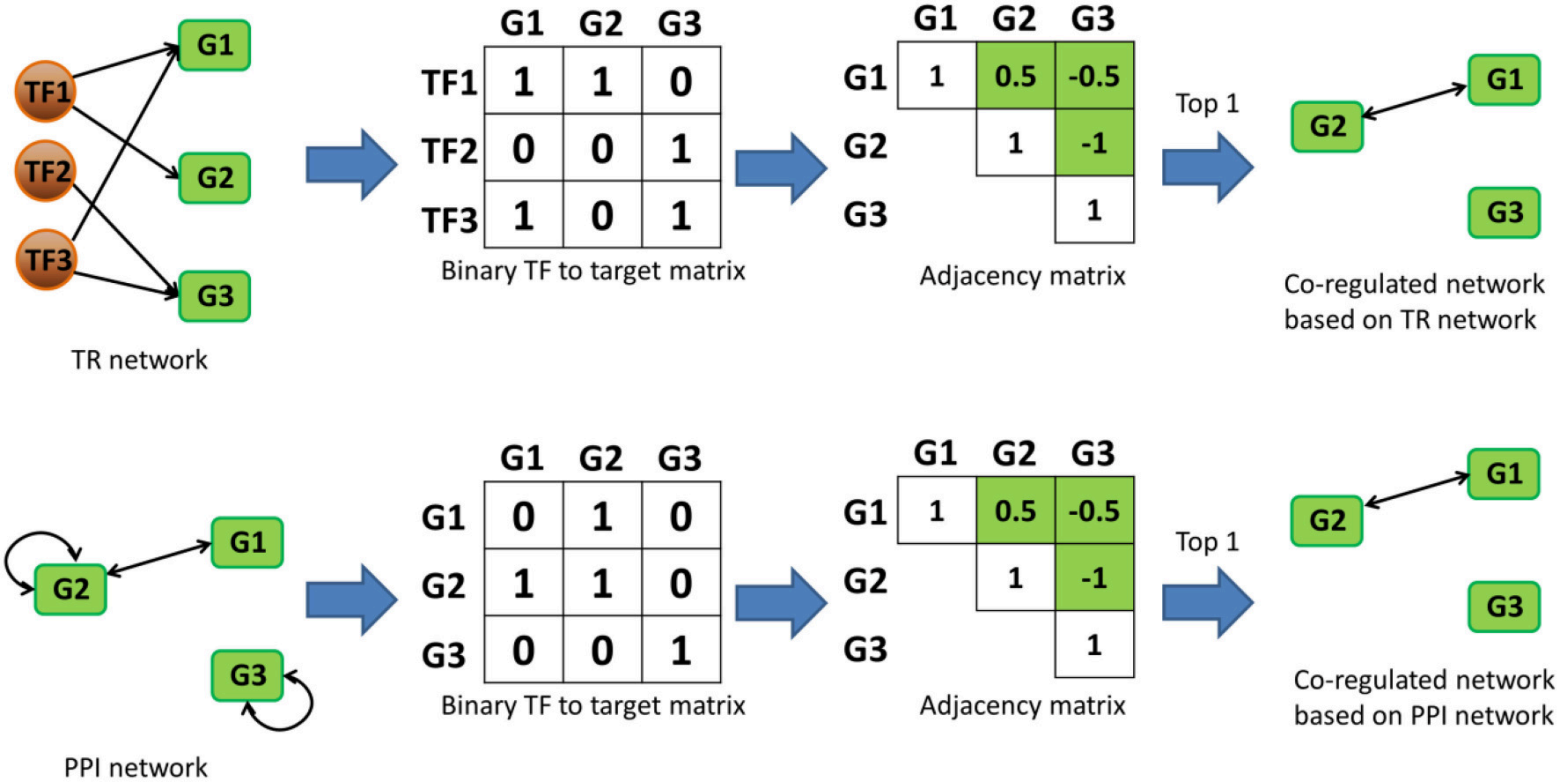
**Toy network case showing how co-regulated networks could be constructed based on TR and PPI networks**. In “binary TF to target matrix,” the ij*th* element is 1 if the i*th* TF is regulating the j*th* target gene. In “adjacency matrixs,” the value of ij*th* element represents the value of Pearson (or Spearman) correlation between the i*th* and j*th* column vectors in the corresponding “binary TF to target matrixs.” The green cells in the “adjacency matrixs” represent the candidate values for the selection of last step, and the diagonal cells are excluded since they are always one. Note that the correlation between two genes is undirected, only half of the remaining “adjacency matrixs” were selected as candidates. “Top 1” in the last step stands for the cutoff and means that only top correlated co-regulated gene pairs were selected in the final co-regulated network.

In this study, our use of the term “co-regulation” was based on the “regulator” similarities of gene pairs in biological networks. Since there's no conventional definition for “regulators” in PPI network, we defined them as the first upstream neighbor proteins that were directly connected by PPIs as starting node of each interactions so they are comparable with regulators in TR networks. Here, “co-regulation” was quantified by calculating the similarity of the “regulators” in each network. We constructed a binary association matrix (i.e., adjacency matrix) for Bnet, Enet and Pnet, and quantified their gene-gene co-regulation similarities by calculating their Pearson correlation coefficients (*r*) [Spearman correlation was also tested and the results were almost identical (Figure S4)]. *r* values were calculated for each target gene pair based on their TF similarities in Bnet and Enet. In Pnet, the first upstream neighbors were used to examine similarities instead of TFs. The top 1 to 5‰ of co-regulated target-target interactions for each network were selected in Bnet, Enet and Pnet, and they were defined as co-regulation networks, namely BCRnet, ECRnet, and PCRnet, respectively. Self-interactions were excluded from the analysis during the co-regulated gene pair selection since their correlation should be one invariantly. The process of constructing co-regulation networks was exhibited for a toy network case in Figure 1.

### Calculation of average co-expression *r* and sensitivity analysis

Throughout this study, average co-expression *r* (ACEr) was used to quantify the network co-expression. For the calculation of ACEr, all self-interactions were excluded to eliminate bias because co-expression and co-regulation measurements from self-interactions were invariably scored as one (or 100%). All ACEr values presented in this study were calculated based on the data from all 1057 microarrays. For sensitivity analysis, the ACEr values were reevaluated by calculating the them for 100 randomly selected conditions from the 1057 microarray data 1000 times.

## Results

### Reconstruction of biological networks and their co-expression

Experiments examining the physical binding properties of TFs and those conducting TF-perturbed expression profiling have different characteristics, and combining those experiments together to infer regulatory interactions has been proposed (Blais and Dynlacht, 2005; Yang et al., 2010). Notably, “expression evidence” in the YEASTRACT database was collected from experiments where the TF was perturbed (e.g., knocked out) after which its targets were identified by scanning genes with significantly changed expression properties. Thus, we favored treating the regulatory interaction information from YEASTRACT separately.

We reconstructed three biological association networks with different biological backgrounds, namely Bnet, Enet, and Pnet, for *S. cerevisiae* based on the YEASTRACT and BioGRID databases (Table S2). Bnet was established from TF-binding experiments, such as chromatin immunoprecipitation (ChIP)-chip, whereas Enet was established using expression evidence. Pnet was formulated from PPIs. There were 5345, 5492, and 5392 genes in Bnet, Enet, and Pnet, respectively, and the number of interactions involved in Bnet, Enet, and Pnet were 35399, 144466, and 245078, respectively. The number of TFs in Bnet and Enet are 169 and 292, respectively. All three networks encompassed the majority of the genes (~95%) included in the microarray data, and their average connectivities were of the same magnitude (a ~7-fold difference between Bnet and Pnet), indicating that the network sizes were comparable to each other.

**Figure 2:**
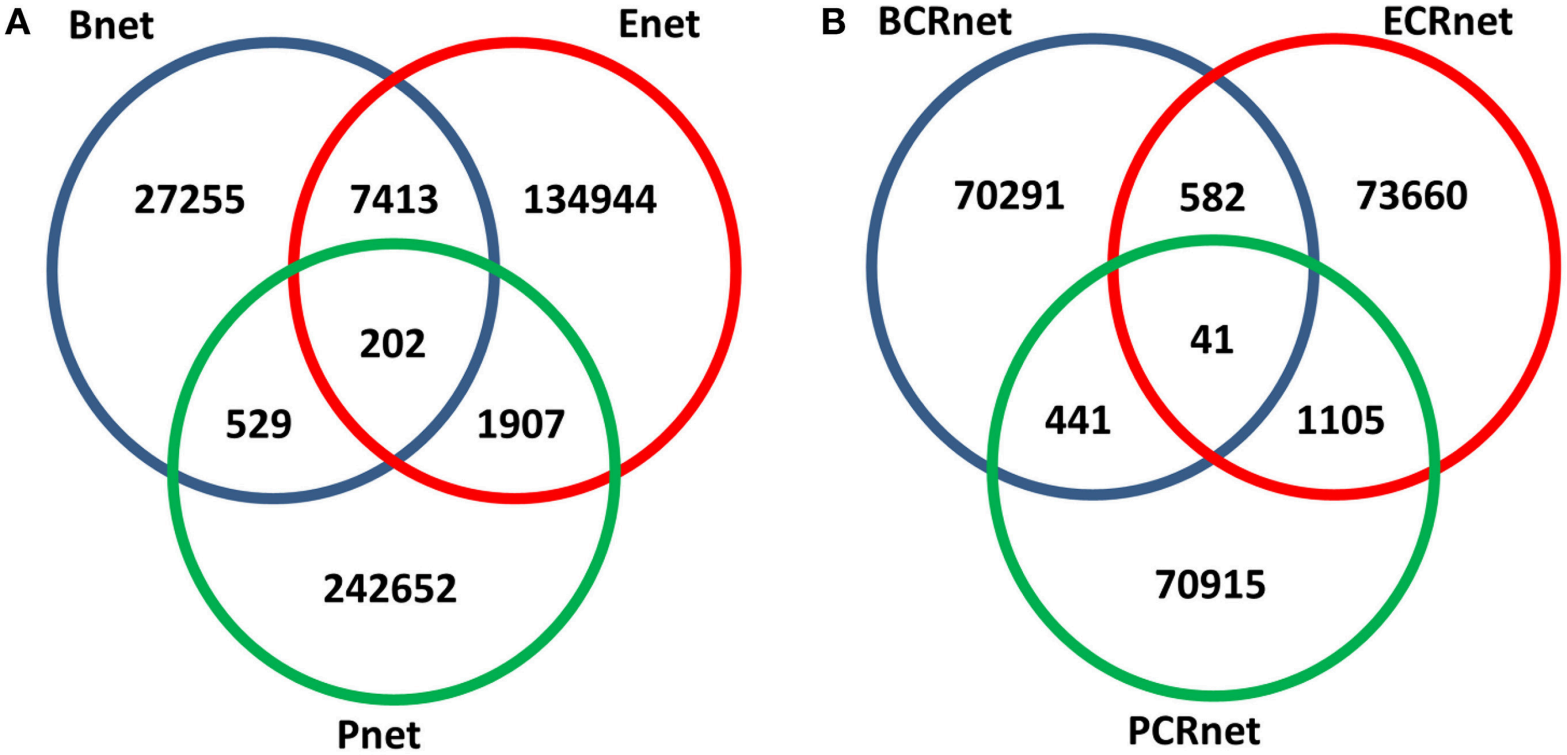
**Overlapped numbers of interactions between networks**. **(A)** Association networks and **(B)** co-regulated networks.

We evaluated the overlaps between the interactions within these three networks and found that the intersections between these three networks were relatively few (Figure 2A). This result suggested that these three networks with different biological backgrounds have their own characteristics. Interestingly, the overlap between Bnet and Enet was relatively small (21.5% for Bnet and 5.3% for Enet) despite the fact that they were both annotated as TR networks and had both originated from the same database. Even if the overlapped TFs and targets were considered, the overlap between them would remain small (Figure S2). This finding implied that the physical binding of TFs to target genes (Bnet) and the summarized effects of specific TF perturbation to expression changes of target genes (Enet) implicated different regulatory events as we proposed. Based on the different biological backgrounds of Bnet/Enet and Pnet and their small overlaps (less than 2%), we assumed that the three networks were different from each other and embedded with different biological information throughout this study.

**Figure 3:**
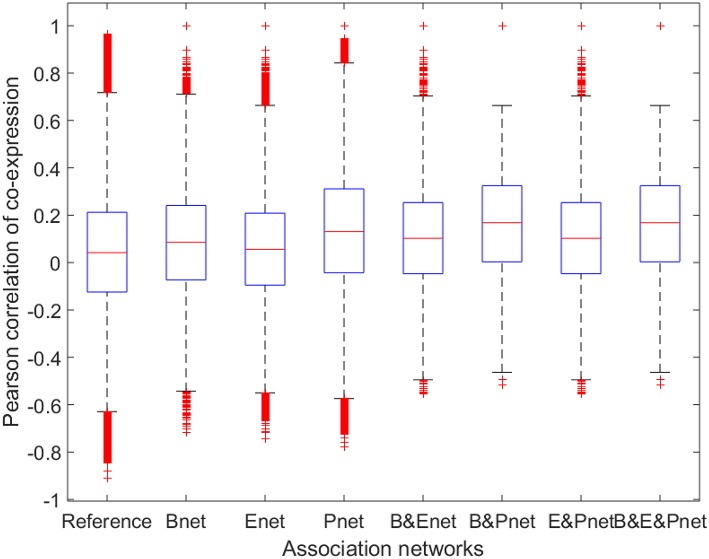
**Boxplot for Pearson correlations of co-expression for all gene pairs involved in reference and association networks**. B&Enet, B&Pnet, and E&Pnet represent the overlaps between Bnet and Enet, Bnet and Pnet, and Enet and Pnet, respectively. B&E&Pnet represents the overlap among Bnet, Enet, and Pnet.

To investigate whether the gene pairs involved in the various biological interactions (regulatory interaction or PPI) were more likely to be co-expressed, we calculated the ACEr values of all gene pairs for the three reconstructed networks separately and compared them to the reference ACEr (Figure 3). The ACEr values for Bnet, Enet, and Pnet were calculated as 0.0841, 0.0568, and 0.1392, respectively. Compared to the reference ACEr, which was 0.0450, the ACEr increases were statistically significant (*P* < 0.001), but their increases were small. The ACEr was higher for Pnet compared with the other two networks, consistent with previous results demonstrating that genes encoding proteins with certain PPI types were more likely to be co-expressed (Jansen et al., 2002). Moreover, when we evaluated the overlapped associations, the ACEr values were further increased compared with the associations from separated networks (except for the intersection between Enet and Pnet whose ACEr values were slightly decreased compared to Pnet). However, these increased ACEr values were also statistically significant (*P* < 0.001) but only slightly increased. These results suggested that although relevant, the gene involvement in both the TR and PPI networks did not necessarily contribute to high co-expression.

### Construction of co-regulated networks and their co-expression

Because the involvement of various biological interactions did not contribute to high gene co-expression, we hypothesized that high co-expression may result from the topological similarities of genes in biological networks. We assumed that if two genes shared similar “regulators” (both in the TR and PPI networks), they tended to be highly co-expressed. To test our premise, we constructed three co-regulated networks (CRnets), including BCRnet, ECRnet, and PCRnet, by selecting the top 5‰ of co-regulated gene pairs from Bnet, Enet, and Pnet, respectively (Table S3). The resulting BCRnet, ECRnet, and PCRnet contained 4520, 4673, and 4541 genes and included 71355, 75388, and 72502 gene-gene interactions, respectively. The overview of the intersections among BCRnet, ECRnet, and PCRnet is presented in Figure 2B. Notably, we generated three CRnets using identical cutoffs, and the resulting CRnets had similar network sizes and connectivities. Despite the similar sizes, the overlaps between the CRnets were small, indicating that the co-regulation networks from Bnet, Enet, and Pnet were different and independent from each other.

**Figure 4:**
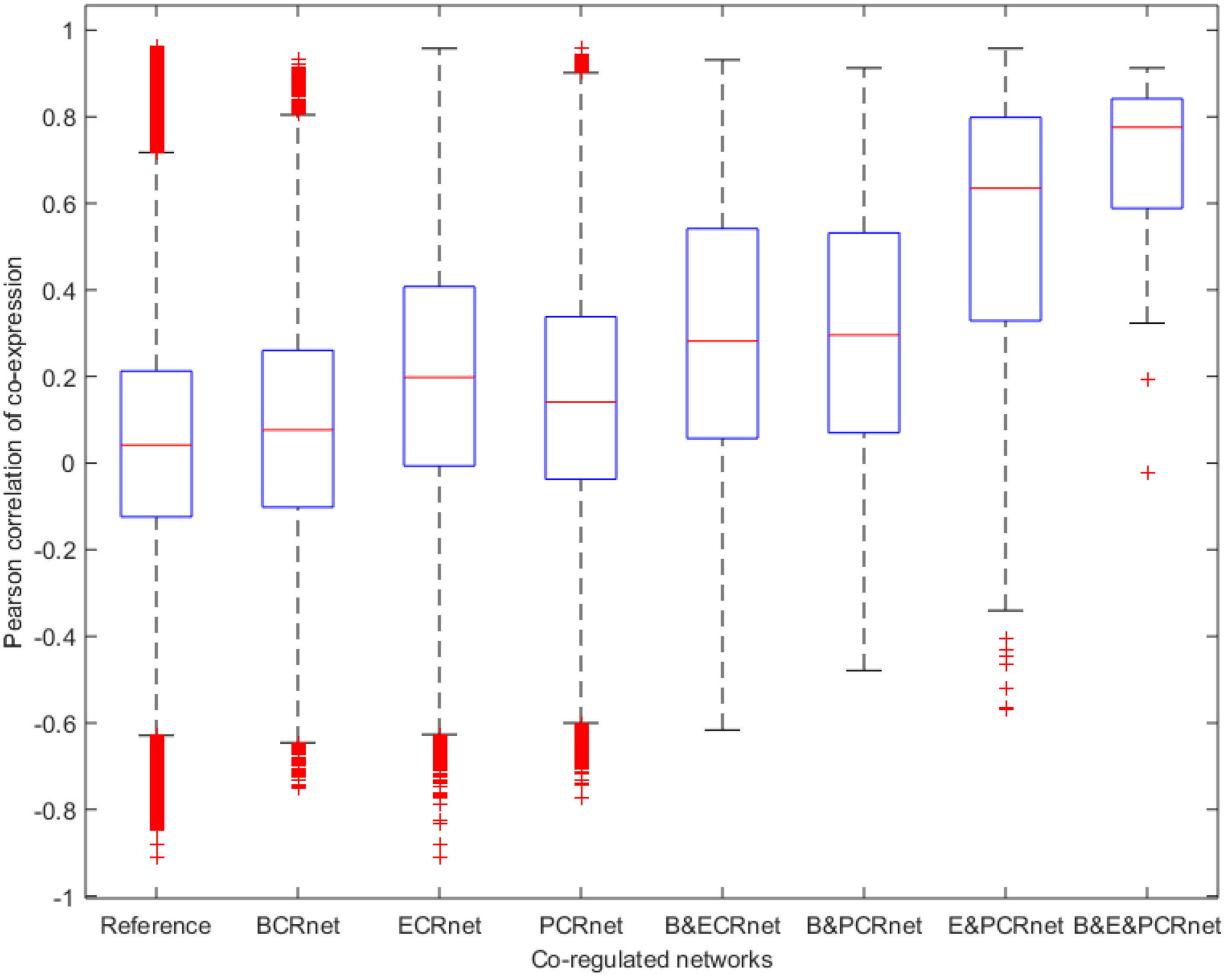
**Boxplot for Pearson correlations of co-expression for all gene pairs involved in reference and co-regulated networks**. B&ECRnet, B&PCRnet, and E&PCRnet represent the overlaps between BCRnet and ECRnet, BCRnet and PCRnet, and ECRnet and PCRnet, respectively. B&E&PCRnet represents the overlap among BCRnet, ECRnet and PCRnet.

**Figure 5:**
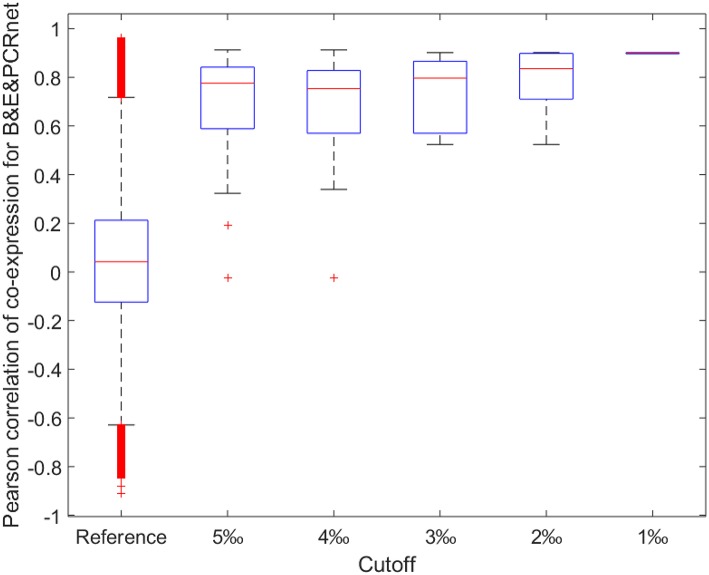
**Boxplot for Pearson correlations of co-expression for all gene pairs involved in reference and shared gene pairs among all three CRnets with different cutoffs**. B&E&PCRnet represent the overlapped gene pairs among BCRnet, ECRnet, and PCRnet.

Next, we calculated the ACEr for each CRnet and evaluated the ACEr of the overlapped interactions among the three networks (Figure 4). Interestingly, we found that the CRnets were generally more likely to be co-expressed compared with their original networks (except for BCRnet, which was slightly less co-expressed). Additionally, significant ACEr increases were noted when the overlapped gene pairs were examined. Intriguingly, when the intersection of all three CRnets was selected, the gene pair ACEr increased to 0.7012, which strongly supported our hypothesis. Furthermore, by changing the cutoffs for CRnet generation, we found that as the cutoff became stricter, the ACEr value increased accordingly. And when the strictest cutoff was selected, the *r* values of the only two gene pairs screened were 0.9012 and 0.8967 (Figure 5). To test the robustness of the positive association between these high correlations and topological similarities, we also performed a sensitivity analysis (see Methods section) for the highly co-expressed gene pairs observed at the intersection of all three CRnets. The results indicated that the calculated ACErs for the overlapped gene pairs of all three CRnets were highly conserved (Figures S3, S4). These results highlighted the consistency of the observed high correlation between co-expression and co-regulation patterns and demonstrated the collaborative outcomes among these biological networks in terms of similarity.

### Gene ontology (GO) term enrichment analysis for the co-regulated gene pairs

To elucidate the underlying biological impact of these co-regulated gene pairs with high similarities, we checked the 41 gene pairs shared in all three CRnets (top 5‰ Table S4). We first examined whether these 41 gene pairs were involved in known biological interactions from Bnet, Enet, and Pnet. Consequently, only 9 of the 41 gene pairs were presented in the interactions of Pnet and none were involved in biological interactions from Bnet or Enet. This finding indicated that we identified 32 genetic associations that were highly co-expressed without previous knowledge of their biological interactions.

**Figure 6:**
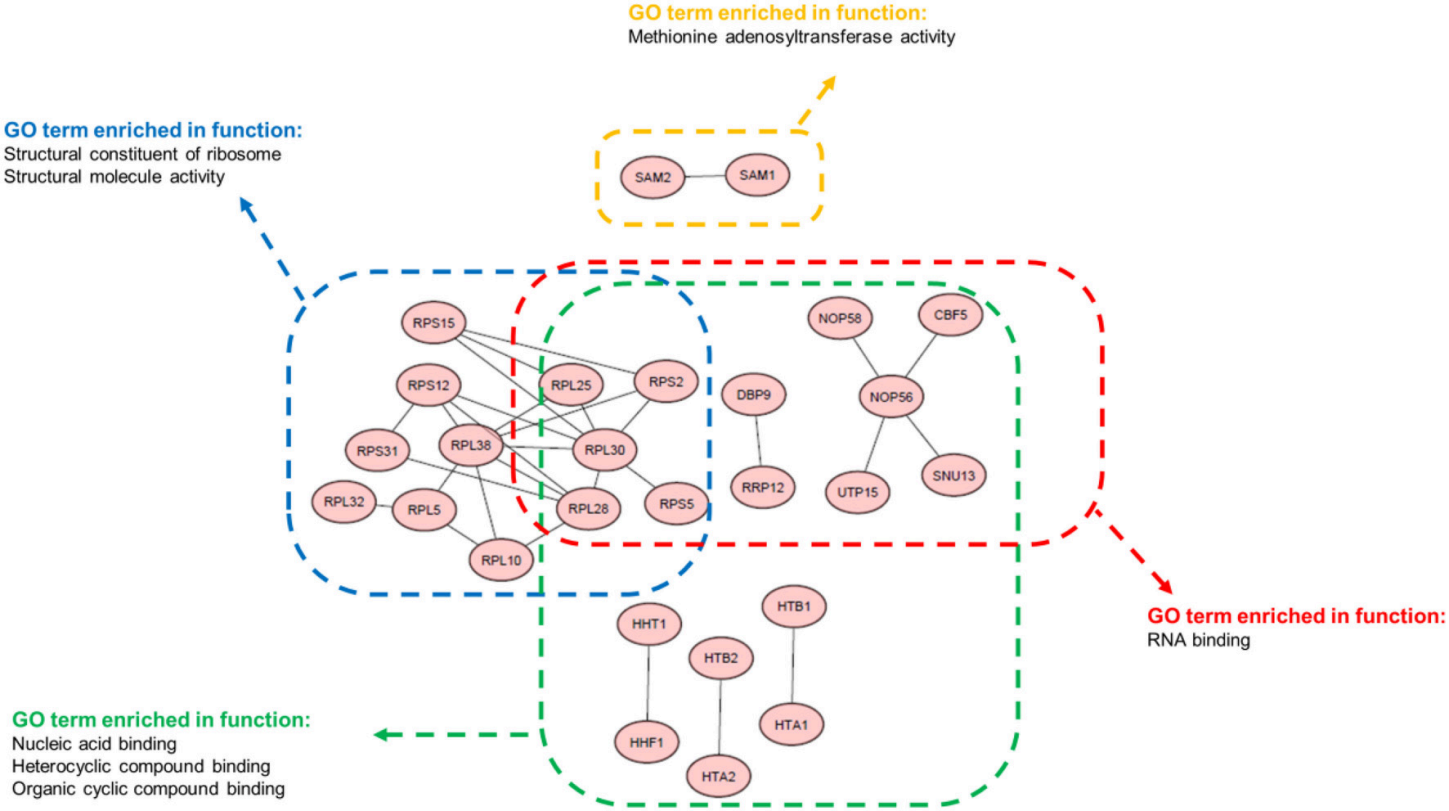
**Functional GO terms enriched among the top correlated gene pairs**. 30 of 41 gene pairs that were enriched in the indicated functional GO terms are displayed. Genes are presented as nodes, and the co-regulated relationships are presented as edges.

Next, we performed a GO enrichment analysis (*P* < 0.01) using the *Saccharomyces* genome database (Cherry et al., 2012) to identify biological processes that were related to co-regulated gene pairs. We found that most gene pairs were either involved in the same process, sharing the same function, or localized to the same cellular component (Table S5). Additionally, these identified co-regulated gene pairs were highly enriched within the GO terms (Figure 6). Interestingly, many of the enriched terms, such as the structural constituents of ribosome-, methionine- adenosyltransferase- and nucleic acid-binding, were related to the regulation of transcription and translation. This finding indicated that the transcriptional and translational processes in yeast were strongly and comprehensively co-regulated. Recent studies have reported that the translational process is crucial to cell physiology, and the ribosome quantity is a key factor that affects proteome allocation (Basan et al., 2015; Hui et al., 2015). Thus, our result is consistent with these studies because it is axiomatic that vital biological processes are highly co-regulated and co-expressed in the evolutionary context. In conclusion, we demonstrated that the highly co-regulated gene pairs identified here are consistently co-expressed, and they share important biologically significant functions.

## Discussion

In this study, we systematically evaluated the individual and combinatory effects of different biological networks on gene co-expression. A comprehensive and unbiased analysis was conducted based on the TR and PPI networks reconstructed here. We demonstrated that co-regulation in biological networks is more relevant to gene co-expression than biological associations. Additionally, we proposed a new method to evaluate the combinatory effects of different co-regulation networks on gene co-expression and found that two genes are highly co-expressed when they are co-regulated in both the TR and PPI networks. Moreover, these co-regulated genes were functionally relevant and involved in vital biological processes. Small-scale studies have demonstrated that co-regulation of gene pairs in the TR network is a key factor contributing to high gene co-expression (Allocco et al., 2004), but a large-scale investigation of co-regulation effects based on topologies provided by regulatory and PPI networks was still lacking. Therefore, our study is an important starting point to study the PPI effect on co-expression and to explore collaborative events between the TR and PPI networks.

It's worth mentioning that the previous study reported a great correlation among genes co-regulated in TR networks (greater than 0.84 when they share more than 50% of their regulators) which is much higher than in our study. However, in our analysis, we also found the co-regulation in TR network is relevant to co-expression (significant but not dramatic), which is actually agreed with the main conclusion of the previous study. And we noted that the previous study only evaluated the co-regulation effect of 2284 genes which are around 40% of our gene set. In addition, they used an outdated version of the TR network, indicating smaller and biased datasets. These would together lead to a much higher correlation probably because of bias. Therefore, our study appeared to be more systematic and robust compared to the previous study.

Our analysis strongly suggests that combinatory effects do exist among biological networks with respect to co-expression, and the genes that are simultaneously co-regulated among the biological networks play important roles in regulating the mRNA expression levels of genes. Interestingly, we found that topological similarities in the PPI network, rather than the interactions themselves, played important roles in modulating gene expression levels. One plausible explanation may be that proteins interacting with the same proteins are functionally related and share similar signaling pathways. Previous genetic epistatic studies have revealed that dysregulated expressions of interacting protein pairs of protein complexes and pathways showed detrimental effects to cell survivals and thus their coding genes might be more likely co-expressed for better genetic fitness (Kelley and Ideker, 2005; Collins et al., 2007). Another possible explanation is that similar PPI interactions of genes may result in similar folding process and/or other post-transcriptional modification of the protein coding genes. And these modification processes may modulate the level of signaling molecules that would normally influence gene regulation.

In our study, we distinguished the regulatory networks of different biological backgrounds separately because we assumed that regulatory interactions inferred from physical binding (i.e., regulations with binding evidence) and genetic perturbation experiments (i.e., regulations with expression evidence) revealed different regulatory events. This assumption is supported by a recent review which discussed that the transcription of genes depends on the combinatory effect of all its binding TFs, and many of them have only a minor effect. While expression evidence is more likely to be the major factor that regulating transcription of genes (Spivakov, 2014). Thus, separating these different networks would help us to make the embedded regulatory messages more apparent. Additionally, when we used merged regulatory networks and tested the shared gene pairs between the co-regulated network of the merged one and the PCRnet, we found that the shared gene pair correlations were substantially decreased (~0.6 when the top 1‰ was selected).

Although these results are informative, several drawbacks to this study should be noted. First, binary networks were used, where the strength of the interactions, albeit important, was not considered. Additionally, there was no negative data for interactions (gene pairs with evidence of no interaction with each other) in publically available databases, impeding a more robust evaluation of co-regulation effects. Therefore, a systematic integration of this information in future studies would improve the correlation between co-expression and co-regulation and would facilitate the interpretation of mechanistic models of gene co-expression.

## Author contributions

CZ participated in design and execution of the study, conducted data analysis and interpretation, and drafted the manuscript. SL participated in design of the study and data interpretation. AM and QH contributed to the design, execution and data interpretation. All authors participated in modifying and editing the manuscript, and the final manuscript had been read and approved by all authors.

## Funding

This work was funded by the National Basic Research Program of China (973 Program) (2012CB721101), National Natural Science Foundation of China (21576089), and China Scholarship Council. This work was also supported by grant from the Knut and Alice Wallenberg Foundation.

### Conflict of interest statement

The authors declare that the research was conducted in the absence of any commercial or financial relationships that could be construed as a potential conflict of interest.

## Supplementary material

Figure S1 **Sketch map of the retrieving of Bnet and Enet from YEASTRACT database**.

Figure S2 **Venn diagram showing the number of shared/unique edges between Bnet and Enet within the same TFs and target genes**.

Figure S3 **Boxplot showing the variation of ACErs for B&E&PCRnet through sensitivity analysis**. Note here the y axis denotes the ACErs.

Figure S4 **Boxplot for Pearson correlations of co-expression for all gene pairs involved in reference and shared gene pairs among all three CRnets with different Spearman correlation cutoffs**. Note that all the CRnets were generated by using Spearman instead of Pearson correlation as cutoff, and the result is identical with those using Pearson correlation (Figure 5).

Table S1 **Normalized microarray data used for the calculation of pairwise gene co-expressions**.

Table S2 **Reconstructed Bnet, Enet and Pnet based on YEASERACT and BioGRID databases**.

Table S3 **Co-regulated networks constructed based on the reconstructed Bnet, Enet and Pnet**.

Table S4 **Detailed list of the identified top co-regulated 41 gene pairs**.

Table S5 **Enriched GO terms of the selected 41 gene pairs in different catogeries**.
