# Supplementary figures and images for: Investigating the Combinatory Effects of Biological Networks on Gene Co-expression
Source: Front Physiol. 2016 May 2;7:160. doi: 10.3389/fphys.2016.00160 (PMC4916787; doi:10.3389/fphys.2016.00160)

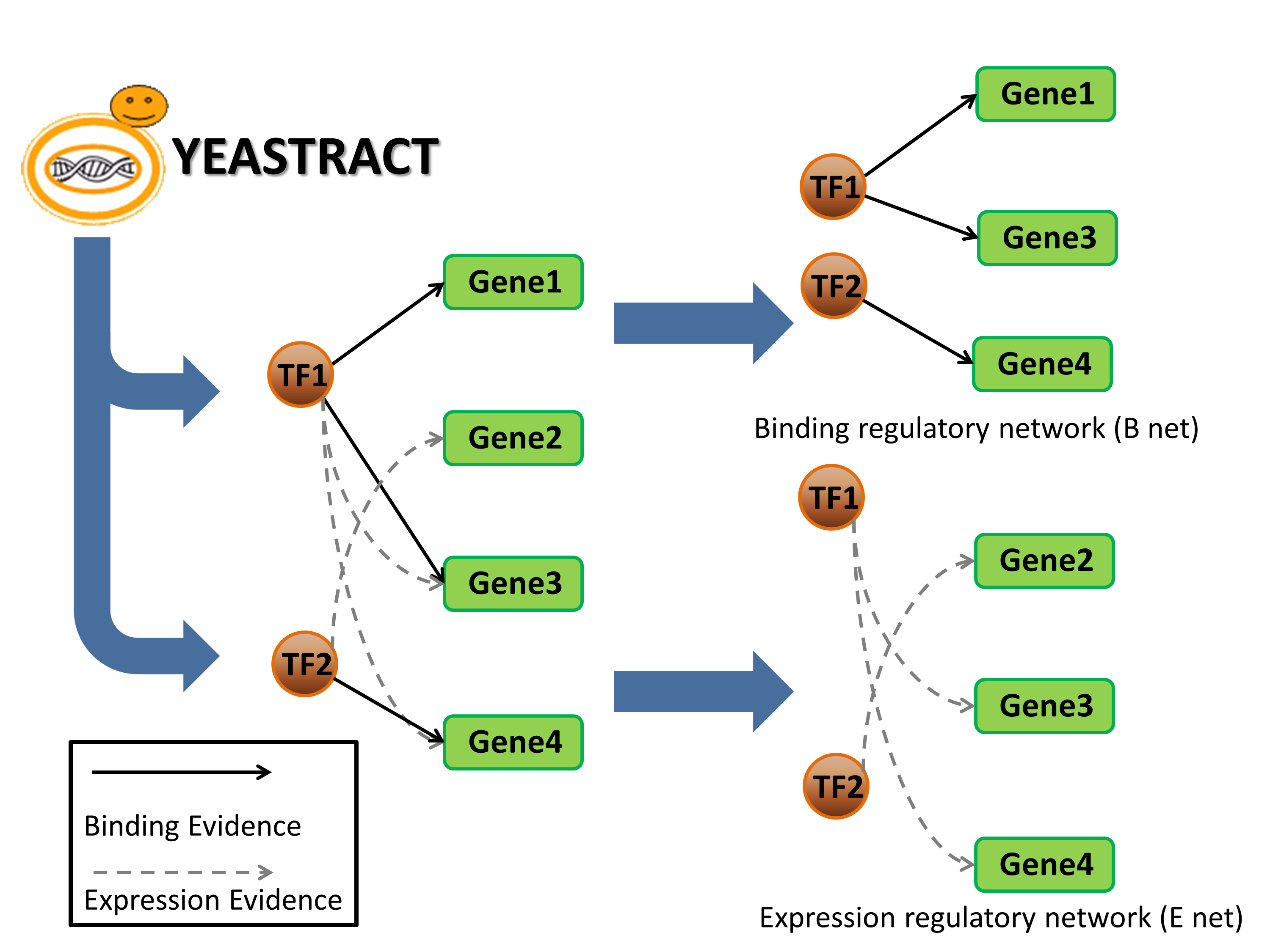

Supplement: Figure S1 — Sketch map of the retrieving of Bnet and Enet from YEASTRACT database. [file Image1.TIF]

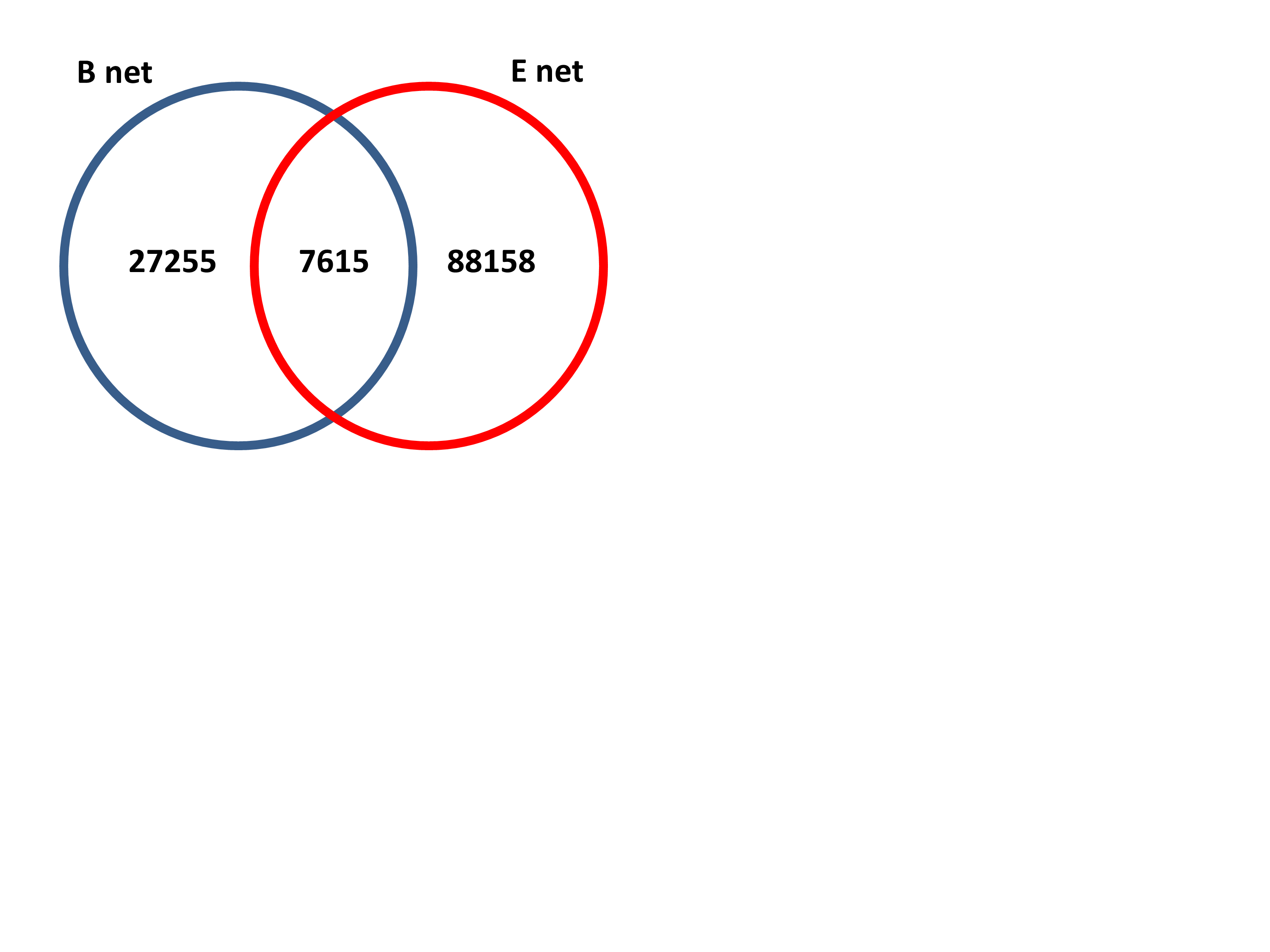

Supplement: Figure S2 — Venn diagram showing the number of shared/unique edges between Bnet and Enet within the same TFs and target genes. [file Image2.TIF]

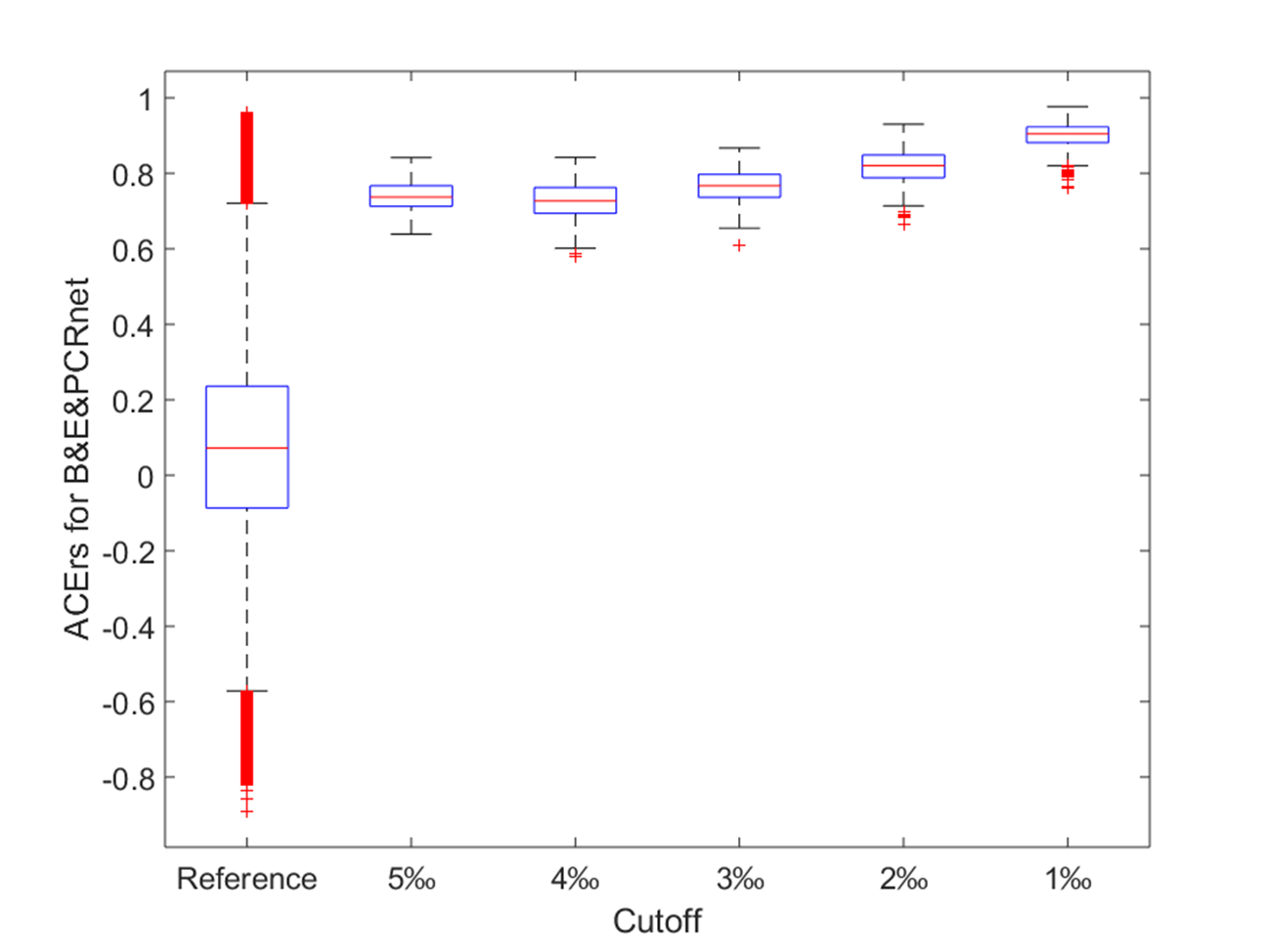

Supplement: Figure S3 — Boxplot showing the variation of ACErs for B&E&PCRnet through sensitivity analysis. Note here the y axis denotes the ACErs. [file Image3.TIF]

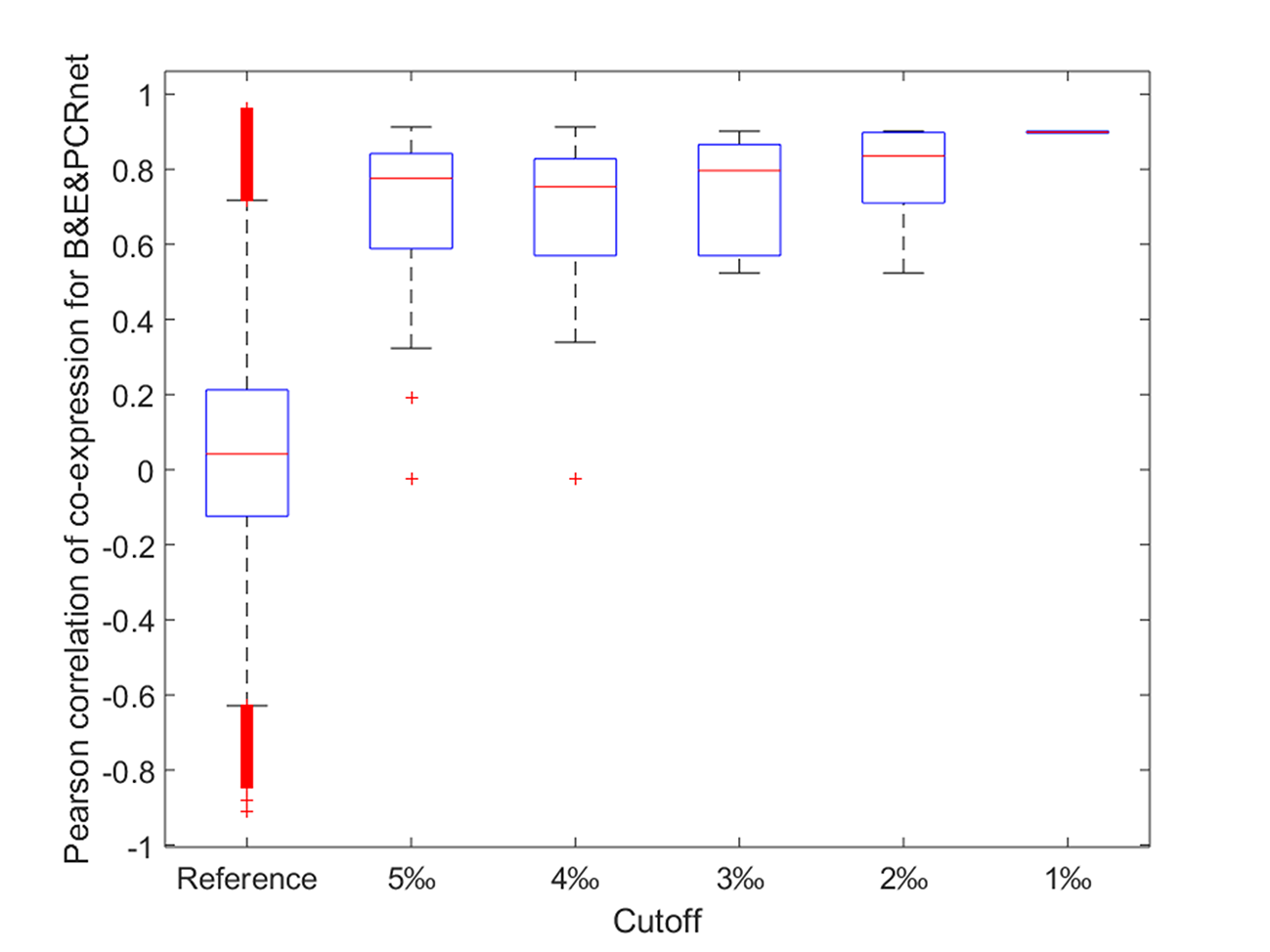

Supplement: Figure S4 — Boxplot for Pearson correlations of co-expression for all gene pairs involved in reference and shared gene pairs among all three CRnets with different Spearman correlation cutoffs. Note that all the CRnets were generated by using Spearman instead of Pearson correlation as cutoff, and the result is identical with those using Pearson correlation (Figure 5). [file Image4.TIF]
